# The role of GLP-1 agonists in perioperative care: a suspension dilemma

**DOI:** 10.1016/j.bjane.2025.844668

**Published:** 2025-08-05

**Authors:** Maisa Ribeiro Araújo, Marcelo Fouad Rabahi, Fabiana Aparecida Penachi Bosco Ferreira

**Affiliations:** a Universidade Federal de Goiás, Faculdade de Medicina, Goiânia, GO, Brazil; b Hospital Israelita Albert Einstein, Goiânia, GO, Brazil

Dear Editor,

In the editorial of this journal, Glucagon-Like Peptide-1 Agonists in Perioperative Medicine: to Suspend or Not Suspend, That Is the Question, several tools for anesthesiologists were discussed to enhance patient safety.^1^ In addition, this letter offers a commentary that synthesizes recent international consensus perspectives to refine perioperative guidance concerning the use of Glucagon-Like Peptide-1 Receptor Agonists (GLP-1RAs) in patients undergoing anesthesia or sedation.

GLP-1RAs and the dual agonists GIP/GLP-1 (Glucose dependent Insulinotropic Polypeptide/Glucagon-Like Peptide-1) are synthetic analogues of a gut-derived incretin hormone secreted after food ingestion. Endogenous hormone has a short half-life of 2–3 minutes and plays a key role in satiety and glucose regulation. In contrast, synthetic GLP-1 RAs such as semaglutide (Ozempic®, Wegovy®) have prolonged elimination profiles – approximately 165 hours (∼7 days) – allowing for convenient once-weekly dosing.^2^ These drugs have revolutionized the treatment of type 2 Diabetes by effectively controlling glycemia with minimal risk of hypoglycemia. Prescription rates have increased further due to their adoption for obesity management, given the significant weight loss observed with the continuous use.^3^ GLP-1RAs confer multisystem organ protection by reducing inflammation, improving endothelial function, lowering lipid levels, enhancing cardiovascular outcomes, and slowing the progression of renal dysfunction in patients with diabetes.^2^^,^^4^

GLP-1RAs dosages are variable and usually titrated gradually due to gastrointestinal side effects. The most common side effects are related to reduced gastric emptying and peristalsis, causing nausea and vomiting, abdominal pain, diarrhea, or constipation. These effects typically occur during the initiation or dose-escalation phase and are self-limited, manifesting across all preparations, short- or long-acting, subcutaneous or oral.^2^^,^^3^ Residual gastric content increases the risk of pulmonary aspiration during general anesthesia/sedation and can result in aspiration pneumonia or chemical pneumonitis.^1^

A clinical practice guideline jointly developed by several American societies, including the American Society of Anesthesiologists, recommends that the decision to continue or withhold GLP-1RA therapy in the perioperative period should be guided by shared decision-making among the patient, anesthesiologist, and prescribing team, with an individualized risk-benefit assessment. The care team should consider variables known to increase the risk of delayed gastric emptying, including dose escalation, higher or weekly dosing regimens, the presence of gastrointestinal symptoms, and medical conditions associated with impaired gastric motility. If the decision is made to withhold GLP-1RA therapy, the optimal suspension interval remains uncertain. However, current recommendations suggest withholding daily formulations on the day of surgery and weekly formulations should be withheld one week prior to surgery. Regardless, all patients should still be assessed on the day of the procedure for gastrointestinal symptoms. Additionally, in patients with suspected delayed gastric emptying, a preoperative liquid diet for at least 24 hours is recommended, similar to bowel preparation used in colonoscopy or bariatric surgery. When clinical concern about retained gastric content exists on the day of the procedure the point-of-care gastric ultrasound can be used to assess aspiration risk.^5^

According to the Brazilian Society of Diabetes, GLP-1RAs should be withheld before a procedure involving general anesthesia or sedation and a specialist should adjust the treatment. Oral or subcutaneous semaglutide should be withheld for 21 days prior to the procedure, and tirzepatide (Mounjaro®) for 15 days, based on the pharmacokinetic principle of three elimination half-lives.^6^

The 2025 consensus of British societies, including Association of Anaesthesists and Royal College of Anaesthesists, recommends continuing GIP/GLP-1 agonists or GLP-1RAs throughout the perioperative period. Their approach emphasizes that the risk of pulmonary aspiration and mitigation strategies should be discussed with the patient using a shared decision-making.^7^

Maselli et al. conducted a retrospective evaluation of 57 patients undergoing Endoscopic Sleeve Gastroplasty without GLP-1RAs discontinuation and found no instances of retained gastric solids on endoscopy. All patients followed a liquid diet on the day prior the procedure and 12 h fasting, emphasizing the potential benefit of preoperative dietary modifications in reducing retained gastric content.^8^

Clinical recommendations from Australian and New Zealand societies emphasize the importance of inquiring patients about the use of GLP-1RAs. For endoscopic procedures, the patients should follow a fluid diet 24 h prior to endoscopy and continue the use of GLP-1RAs. For non-endoscopic procedures the focus remains on patient engagement in risk assessment and procedural planning. All patients taking these drugs should be considered non-fasted. The point-of-care gastric ultrasound should be considered for risk stratification to determine the qualitative and quantitative content of stomach before the anesthesia. Extending the fasting time is not recommended given the current lack of evidence and the absence of gastrointestinal symptoms does not exclude retained gastric content, but the presence of gastrointestinal symptoms may be associated to retained gastric content.^9^

Pharmacokinetic and clinical data indicate that short interruptions of long-acting GLP-1RAs (one half-life) may not be sufficient to complete drug clearance. Currently, no data available on gastric emptying from residual GLP-1RAs levels and prolonged interruptions (four or five half-lives) could be impractical, clinically harmful, and inconsistent with a patient-centered approach.^3^

Tracheal intubation using cuffed tubes is the best method for airway protection against the aspiration, however, it is not foolproof. Accumulated secretions may bypass the cuff into the trachea, especially in cases of large-volume regurgitation, in patients positioned in Trendelenburg during the procedure or other reflux-facilitating conditions, such obesity or laparoscopic procedures. Point-of-care gastric ultrasound has emerged as a critical tool for aspiration risk stratification in this context. As discussed above, there is insufficient data on the residual effects of GLP-1RAs on gastric emptying, so it would be unsafe to assume that standard fasting protocols ensure gastric emptying, regardless of the drug suspension time. Even if regurgitation does not occur at the time of anesthetic induction, it may still occur during the procedure, patient positioning, extubation or in the post-anesthesia recovery room, sometimes without the medical team’s awareness, leading to postoperative pulmonary complications and delaying the suspicion of aspiration pneumonia.

The current literature increasingly supports the continuation of GLP-1RAs due to their clinical benefits. However, there remains a lack of robust data on the optimal preoperative dietary strategy to ensure complete gastric emptying. Until further evidence becomes available, two key practices should be integrated into perioperative protocols: implementation of an institutional protocol aimed at systematically screening patients for GLP-1RA use, and incorporation of gastric ultrasound. These tools enable individualized risk stratification and management, prioritizing patient safety in the perioperative setting, independent of drug discontinuation status.

## Conflicts of interest

The authors declare no conflicts of interest.
